# Ureteral wall thickness as a predictor for non-invasive treatment success for steinstrasse. Can we save time?

**DOI:** 10.1007/s00345-024-04874-w

**Published:** 2024-03-13

**Authors:** M. A. Elbaset, Diaa-Eldin Taha, Marwan Anas, Ahmed Elghareeb, Rasha T. Abouelkheir, Rawdy Ashour, K. Z. Sheir, Yasser Osman

**Affiliations:** 1 Urology Department, Urology and Nephrology Center, Mansoura University, Mansoura, Egypt; 2 Urology Department, Horus University, New Damietta, Egypt; 3 Urology Department, KafrELshiekh University, KafrELshiekh, Egypt; 4 Radiology Department, Urology and Nephrology Center, Mansoura University, Mansoura, Egypt

**Keywords:** Shockwave lithotripsy (SWL), Medical expulsive therapy (MET), Ureteral wall thickness (UWT), Stone, Steinstrasse (SS)

## Abstract

**Purpose:**

We aimed to define factors affecting the non-invasive overall treatment success (medical expulsive therapy (MET) ± shock wave lithotripsy (SWL)) for uncomplicated ureteral steinstrasse (SS) clearance.

**Methods:**

We retrospectively evaluated consecutive patients who underwent SWL for renal stones between 2017 and 2021. Patients with uncomplicated SS were included. All patient’s demographic and radiological data, e.g., age, gender, pre-SWL stenting, SS site, type, leading stone size in widest diameter (< 10 mm and ≥ 10 mm), ureteral wall thickness (UWT) in mm against the leading stone were collected. If SS was diagnosed, medical treatment was given for 4 weeks. In case of MET failure, either SWL for the leading stones + MET or direct URS was done. Non-invasive treatment success (SFR) was considered if complete clearance of SS occurred with no complications or the need for invasive intervention.

**Results:**

A total of 145 patients were included with mean age of 45.9 ± 12.4 years. SFR in case of MET only occurred in 27.9%. Complications happened in 26 patients (17.9%). Non-invasive treatment SFR was achieved in 78 patients (53.8%) totally where SS type I, leading stone size ≤ 10 mm type and decreased UWT around the leading stone increased treatment success.

**Conclusion:**

Ureteral wall thickness is an important factor predicting SS management success. Besides the decreased UWT, non-invasive management should be offered for type I SS with leading stone ≤ 10 mm.

**Supplementary Information:**

The online version contains supplementary material available at 10.1007/s00345-024-04874-w.

## Introduction

One of the most effective minimally invasive treatment for sizable renal stones is extracorporeal shock wave lithotripsy (SWL) [1]. Fragments clearance depends on ureteral motility, edema and spasm in addition to fragment size and site [2]. Aggregation of stone fragments in the ureter following SWL is called steinstrasse (SS) [3]. It is usually a transient complication [4]. In case of associated complications; progressive hydronephrosis, urinary tract infection (UTI), increased loin pain, prompt intervention is recommended to reduce morbidity. Percutaneous nephrostomy (PCN) insertion is the treatment of choice as placement of ureteric stent is usually unsuccessful.

On the other hand, in case of uncomplicated SS, there is no solid guidelines about optimal treatment selection [1, 5]. Delay to the optimal treatment could lead to acute pyelonephritis and sepsis. In a previous study, 5% of patients with SS developed infected hydronephrosis within 4 weeks [6]. So, the reasonable treatment selection can diminish the hazards of progressive ureteral obstruction and complications. Modalities of intervention ranges from non-invasive techniques as conservative management including medical expulsive treatment (MET) and repeated SWL to the leading stone to invasive techniques, e.g., endoscopic intervention and finally open surgery [3].

Use of α-blockers in case of SS is a matter of controversy. Some reported non-significance of adding α-blockers while others reported its efficacy in increasing SFR [7–10]. Combined α-blockers with SWL, improve stone clearance theoretically by increasing both ureteral flow and pressure gradient above the stone [11]. It was reported that SWL for the leading stone was a successful modality for failed expectant treatment in case of SS [6].

Dealing with the non-invasive SS treatment, stone factors were merely reported ignoring other ureteral characteristics accountable for stone clearance and the best treatment selection. So, we aimed in this study to identify the predictors for non-invasive management success for ureteral SS clearance.

## Materials and methods

### Patients

We retrospectively evaluated consecutive patients who underwent SWL for renal stones between January 2017 and October 2021. Patients with uncomplicated SS were included. Patients less than 18 years, missed follow-up, patients who underwent immediate ureteroscopy (URS), complicated SS, patients who were followed up by KUB or US only after the first session, congenial renal malformations, patients who underwent URS before SWL sessions with or without JJ stent fixation, solitary renal units and anatomical obstruction distal to the stone were excluded.

All patient’s demographic data were collected, e.g., age, gender, BMI was classified as non-obese (BMI ≤ 30 kg/m^2^) and obese (> 30 kg/m^2^), presence of ureteral stenting pre-SWL. Radiological data were assessed using spiral multi-slice NCCT, e.g., site of SS (either pelvic, iliac or lumbar), leading stone size in widest diameter (< 10 mm and ≥ 10 mm), leading stone density in HU, type of SS (type I: composed of fine particles of 2 mm, type 2: stone fragments with leading fragments of 4–5 mm and type 3 composed of large fragments)[3], ureteral wall thickness (UWT) [12, 13] in mm against the leading stone.

### Procedure

Using Dornier MedTech GmbH electromagnetic lithotripter, Germering, Germany, SWL sessions were performed under the supervision of a single expert urologist. Power ramping up to 16 kV with rate of 80 shocks/min and a maximum of 3000 shocks per session were used. During treatment, stones were targeted with fluoroscopy and/or ultrasound at regular intervals through the procedure for follow-up. After the SWL session, MET in form of α-blocker (Tamsulin^®^ 0.4 mg capsule once daily) was given to all cases for 2 weeks.

If SS was diagnosed by low-dose NCCT, treatment was continued for another 2 weeks. In case of no stone expulsion, either SWL of the leading stones combined with MET or direct URS was done according to the surgeon’s opinion. SWL sessions were done by 2-week interval with a maximum of three sessions. If there is no stone expulsion, patients are referred to URS. Patients with complications were subjected to urgent invasive treatment. Non-invasive treatment success was considered if complete clearance of SS fragments occurred with no associated complications or the need for invasive intervention.

### Outcomes

*Primary outcome* was to define factors affecting the non-invasive overall treatment success (MET ± SWL) for uncomplicated ureteral SS fragments clearance post-SWL. *Secondary outcome* was to define factors affecting the success in group managed by MET alone.

### Statistical analysis

Patient’s demographics and clinical data were compared using the Chi-square test, Mann–Whitney test or independent sample t-test according to the situation. Univariate and logistic regression analyses were performed to identify factors that contribute treatment success. Receiver operating characteristic (ROC) curve to identify the cut-off values for the best sensitivity and specificity for significant variables in univariate was performed. All statistical analyses were performed using SPSS version 21 and *p* < 0.05 was considered statistically significant.

## Results

A total of 226 patients were initially included in the study, whereas 81 patients were excluded as they did not met the inclusion criteria. Finally, 145 patients were included in the study and the mean age was 45.9 ± 12.4 years. The majority of included patients were males in gender. SS were formed in 104, 33 and 8 patients post the 1st, 2nd and 3rd SWL session, respectively. All patients received MET for 4 weeks with SFR of 27.6% (40 patients). Twenty-six patients developed complications and received urgent interventions (17.9%). Other patients were managed by SWL for the leading stone in addition to MET (54.5%). In the latter group, SFR was observed in 38 patents (48%) (Supplementary Fig. 1).

**Fig. 1:**
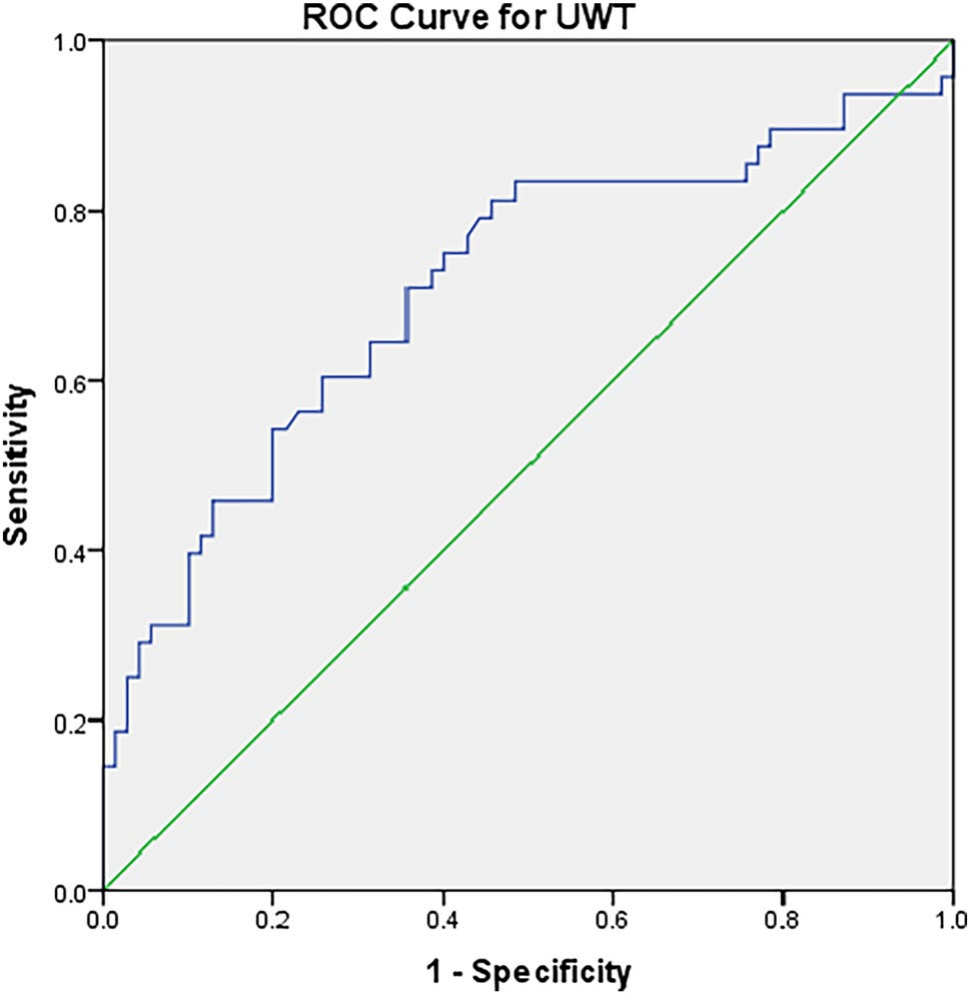
ROC curve for UWT as a predictor for non-invasive management success for SS. AUC: 0.71 and *P* < 0.0001 and cut-off value for UWT is 3.2 mm with sensitivity and specificity is 72% and 59%, correspondingly

Total treatment success (SFR) (MET ± SWL) was achieved in 78 patients (53.8%). Univariate analysis was done to evaluate factors affecting the non-invasive overall success for complete ureteral SS clearance. We found that type I SS, leading stone size ≤ 10 mm and decreased UWT around the leading stone were associated with increased success with *P* = 0.002, 0.007 and < 0.0001, correspondingly. Neither the stone site nor the mode of treatment was a predictor for treatment success. These variables was sustained significantly in multivariable analysis. They increased the risk for treatment success by 2.7, 10.4 and 1.5 times *P* = 0.04 and < 0.0001, respectively (Table 1). We found that a UWT of 3.2 mm was the best cut-off value responsible for treatment success using ROC curve (Fig. 1).

**Table 1:** predictors for non-invasive treatment (MET ± SWL) success in SS post-SWL

Variable	Total patients *N* = 145	Treatment success *N* = 78	Treatment failure *N* = 67	*P* value	Multivariate analysis OR (95%CI) *P* value
Age in years (mean ± SD)^a^	45.9 ± 12.4	43.97 ± 12.87	48.2 ± 11.6	0.3	
Gender (no. of pts)^b^					
Male	109 (75.2)	57 (73.1)	52(77.6)	0.5	
Female	36 (24.8)	21 (26.9)	15(22.4)		
Obesity (no. of pts)^b^					
Non-obese	72 (49.7)	44 (56.4)	28 (41.8)	0.07	
Obese	73 (50.3)	34 (43.6)	39 (58.2)		
Presence of ureteral stent (no. of pts)^b^					
No	133 (91.7)	74 (94.9)	59 (88.1)	0.1	
Yes	12 (8.3)	4 (5.1)	8 (11.9)		
Mode of treatment (no. of pts)^b^					
MET only	66 (45.5)	40 (51.3)	26 (38.8)	0.1	
MET + SWL	79 (55.5)	38 (48.7)	41 (61.2)		
Site of stones (no. of pts)^b^					
Pelvic	75 (51.7)	40 (51.3)	35 (52.3)	0.7	
Iliac	19 (13.1)	9 (11.5)	10 (14.9)		
Lumbar	51 (35.2)	29 (37.2)	22 (32.8)		
Type of steinstrasse (No. of pts)^b^					
Type I	104 (71.7)	65 (83.3)	39 (58.2)	0.002	**2.7 (1.05–7.52) 0.04**
Type II	31 (21.4)	11(14.1)	20 (29.9)		
Type III	10 (6.9)	2 (2.6)	8 (11.9)		
Stone size (no. of pts)^b^					
< 10 mm	114 (78.6)	68 (87.2)	46 (68.7)	0.007	**10.4 (3.02–18.1) < 0.0001**
≥ 10 mm	31 (21.4)	10(12.8)	21 (31.3)		
Ureteral wall thickness (median & range)^c^	2 (1–11)	2 (1–6)	4 (1–11)	< 0.0001	**1.5 (1.3–2.89) < 0.0001**
Hounsfield unit of the stones (Mean ± SD)^a^	830 ± 291	825 ± 294	835 ± 290	0.8	

Bold values mean statistically significance

*No. of pts* number of patients

^a^independent sampled t test

^b^Chi-square test &

^c^Mann–Whitney test

In patients who were managed by MET only, treatment success was observed in 27.5% within a median (range) of 3 (1–4) weeks. In univariate analysis, stone size ≤ 10 mm and decreased UWT against the leading stone fragment were significant parameters increased the chance of treatment success with *P* = 0.003 and 0.001, respectively (Table 2). In multivariate analysis, stone size ≤ 10 mm in addition to decreased UWT around the leading stone were predictors for treatment success with *P* = 0.02 and 0.01, respectively (Table 2).

**Table 2:** Predictors for treatment success for MET alone in SS post-SWL

Variable	Treatment success *N* = 40	Treatment failure *N* = 105	*P* value	Multivariate analysis OR (95%CI) *P* value
Age in years (mean ± SD)^a^	44.1 ± 11.2	46.6 ± 12.8	0.1	
Gender (no. of pts)^b^				
Male	30(75)	79 (75.2)	0.9	
Female	10(25)	26 (24.8)		
Obesity (no. of pts)^b^				
Non-obese	22 (55)	50 (47.6)	0.4	
Obese	18 (45)	55 (52.4)		
Presence of ureteral stent (no. of pts)^b^				
No	39 (97.5)	94 (89.5)	0.2	
Yes	1 (2.5)	11 (10.5)		
Time to clearance in weeks (mean ± SD)^a^	2.5 ± 1.2	3.9 ± 1.1	0.1	
Site of stones (no. of pts)^b^				
Pelvic	19 (47.5)	56 (53.3)	0.7	
Iliac	5 (12.5)	14 (13.3)		
Lumbar	16 (40)	35 (33.3)		
Type of steinstrasse (no. of pts)^b^				
Type I	31 (77.5)	73 (69.5)	0.6	
Type II	7 (17.5)	24 (22.9)		
Type III	2(5)	8 (7.6)		
Stone size (no. of pts)^b^				
≤ 10 mm	38 (95)	76 (72.4)	0.003	**7.85 (1.3–14.5) 0.02**
> 10 mm	2 (5)	29 (27.6)		
Ureteral wall thickness (median & range)^c^	2 (1–3)	3 (1–7)	0.001	**3.95 (1.6–11.92) 0.01**
Hounsfield unit of the stones (mean ± SD)^a^	745 ± 284.6	853 ± 290	0.9	

Bold values mean statistically significance

*No*. *of pts* number of patients

^a^Independent sampled t test

^b^Chi-square test

^c^Mann–Whitney test

## Discussion

Actually, the treatment of ureteral stone fragments post-SWL for renal calculi is not a bit altered from that of ureteral stones. After SWL, SS has been subclassified according to the lead fragment size in addition to fragments size above the leading stones to 3 main categories [3]. The incidence of SS has been observed in 2%-20% in KUB radiography with increased the risk in case of increasing stone burden pre-SWL [3, 6, 7, 11].

Usually conservative management is more cost-effective and simple treatment than active stone treatment. In this context, Fedullo et al. [8] reported that 65% of patients with SS could pass fragments expectantly without medications. On the other hand, Coptcoat et al. [3] concluded that spontaneous passage in type III SS is rare and there is no benefit for waiting, early intervention is warranted in such cases. Phukan et al. [14] in another study reported that SFR was achieved by conservative management in 47% of included patients. Intervention (either PCN placement, SWL or URS) was needed in case of type III SS [14]. The authors recommended early intervention in this type of SS without observation [14]. Goyal et al. [6] reported that conservative treatment should be offered only for type I SS. In case of type II and Type III, 90% usually not responding to the conservative management for 4 weeks and recommended early SWL for the leading stones if size exceeded 5 mm [6].

In our study, SFR was noted in 53.8% of patients managed by non-invasive approach. We found that presence of leading stone size ≤ 10 mm, decreased UWT ≤ 3.2 mm around the leading stones in addition to type I SS were predictors for treatment success. In 80% of patients with type III SS, failure to clear stone fragments was noted. This latter finding is similar to the previous findings that early endoscopic intervention is needed for type III SS without any attempt for conservation [3, 6, 14].

Passage of SS fragments is affected by factors such as ureteral smooth muscle spasm, in addition to edema of the ureteral wall against the leading stones [7]. So, ureteral wall relaxation in the region of the stone fragment is considered to be an important factor promoting stone passage. Based on that, the use of α-blockers can facilitate stone fragments passage through ureteral relaxation [15]. But the results of α-blockers use in SS specifically is contradictory with the need to be optimized for different case scenarios.

Resim et al. [7] reported that tamsulin did not significantly improve the SFR (75% and 65% spontaneous resolution, respectively; *P* = 0.05). Bhagat et al. [10] documented the same results as tamsulin did not add benefit in increasing SFR in small stones less than 5 mm but it significantly increase retained ureteral stones with larger stone particles. On the other hand, Moursy et al. [9] in a randomized trial concluded that α-blockers use significantly increase the SFR in case of SS compared with expectant therapy.

In our study, in patients who were managed by MET in the form of α-blockers SFR was achieved in 27.5% of patients. Leading stone size ≤ 10 mm was found to increase the possibility of stone fragments expulsion by 7.8 times. Based on the guidelines, in uncomplicated ureteral stones ≤ 10 mm, conservative treatment including observation or MET should be offered for 4–6 weeks [1]. During MET, stone index profile, e.g., stone size is an important clinical factor predicting SFR in case of ureteral stones [16]. SFR can be achieved in 68% and 47% of patients with ureteral stones < 5 mm and ≥ 5 mm to < 10 mm ureteral stones during conservative management [1]. In our study, SFR was achieved in 95% of patients with leading stone size ≤ 10 mm. Improvement of treatment results could be done if we consider additional ureteral index factor like ureteral wall edema.

UWT recently was proposed to predict failure of SWL, retrograde ureteral stent placement in addition to failure to manipulate stones during URS [12, 13, 17, 18]. Increased UWT indicate the presence of impacted ureteral stones. Stone impaction occurred due to ureteral local inflammation, polyps and edema when stone fragments remain at the same location for a prolonged time period [18]. Yoshida et al., reported that measuring UWT by NCCT before starting treatment can be a good indicative marker for stone passage < 10 mm during MET [19]. In our study, decreased UWT ≤ 3.2 mm was a predictor of overall non-invasive treatment success in addition to success of each type of treatment solely.

Since kim et al. [11] introduced the idea of repeated SWL for in case of SS aiming to disintegrate the lead stone fragment causing obstruction in addition to mechanically loosen the small fragments proximal to it. Authors in this study documented that repeated SWL for the leading stone achieved SFR in 88.9% out of 18 patients and only 2 patients required surgical intervention [11]. In our study, only 48.1% of patients were stone free after combined treatment. Only 4 (19%) patients out of total 21 patients in both types II and III managed by combined approach achieved complete stone clearance. We could explain these results by the increased UWT in group of patients who failed to clear stone fragments around the leading stone. We could notice in addition that UWT around the leading stone in patient who failed to clear stone fragments is more in patients managed combined treatment in comparison with the same group in conservative management alone.

The study was limited by small sample size with a retrospective nature with inherent bias in patient’s selection who were treated by combined approach or direct URS. Failure to assess the quality of life (QoL), lack of radiation exposure estimation and financial costs calculation were other limitations.

## Conclusion

Ureteral wall thickness is a predictive tool expecting non-invasive treatment success (conservative (medical expulsive therapy only) and combined (medical expulsive therapy + shockwave lithotripsy for the leading stones)) in case of ureteral steinstrasse post-shockwave lithotripsy and should be taken in consideration. Besides the decreased ureteral wall thickness around the leading stone ≤ 3.2 mm, non-invasive management generally in steinstrasse should be offered for leading stone ≤ 10 mm and type I steinstrasse to achieve the best stone free rate. In case of conservative management, treatment success was noted in nearly one third of cases in a median of 4 weeks depending on decreased ureteral wall thickness and smaller leading stone size ≤ 10 mm. More prospective trials are recommended to confirm our findings.

## Supplementary Information

Below is the link to the electronic supplementary material.

Supplementary file1 (DOCX 31 KB)

## Data Availability

Data and materials are not publicly available, but are available by the corresponding author upon reasonable request.
