# Supplementary material for: Ureteral wall thickness as a predictor for non-invasive treatment success for steinstrasse. Can we save time?
Source: World J Urol. 2024 Mar 13;42(1):151. doi: 10.1007/s00345-024-04874-w (PMC10937774; doi:10.1007/s00345-024-04874-w)
Supplement: Supplementary file 1 — Supplementary file1 (DOCX 31 KB) [file 345_2024_4874_MOESM1_ESM.docx]

**Figure (1): Study flow chart.**

**226 were initially included with SS**

**81 patients were excluded:**

**Obstructed infected kidney=4**

**Patients managed by immediate URS= 77**

**145 patients were managed by MET for 4 weeks**

**Complications =26**

**Obstructed infected kidney=6**

**Patients underwent URS=20**

**SWL + MET=79**

**Treatment success= 40**

**Complications = 41**

**Obstructed infected kidney=5**

**Patients underwent URS=36**

**Treatment success = 38**
